# Theft of Gramsci? On the radical right, radical left, and common sense

**DOI:** 10.1007/s10624-022-09681-6

**Published:** 2023-01-11

**Authors:** Agnieszka Pasieka

**Affiliations:** Department of Social and Cultural Anthropology, University of Vienna, Vienna, Austria

**Keywords:** Radical right, Radical left, Gramsci, Common sense, Fascism

## Abstract

Drawing on ethnographic research with radical right-wing activists in Italy and Poland, my article reflects on the ways in which the Gramscian framework may enhance our understanding of the present-day political landscape. Gramsci’s role in the article is threefold. First, since he was a keen observer of fascist developments, I relate his observations on fascism and inquire into their relevance for understanding the rise of the far right today. Second, I explore the agendas of the movements I studied through the Gramscian lens. Inspired by the special issue’s editors, I examine the extent to which Gramsci’s concept of “common sense” is helpful for analyzing contemporary far-right activism. Third, I relate my own ethnographic observation to analyses of a broader terrain of far-right politics to shed light on the phenomenon of “far-right Gramscianism.” Bringing together all these observations on the radical right, “common sense” and Gramsci’s legacy, I reflect on the complex interrelationship between the radical right and the radical left.

“I hate the indifferent. I believe that living means taking sides.

Those who really live cannot help being a citizen and a partisan.

Indifference and apathy are parasitism, perversion, not life.”

*Antonio Gramsci, August 1916*

## Introduction

“You need to read Gramsci,” Ula exclaimed with a smile, interrupting Krystian, who was in the midst of a heated political debate.

“I know his theory, but I don’t buy it,” Krystian replied, annoyed by her interruption.

I was with Krystian, the founder of a nationalist-socialist movement, and Ula, a sympathizer with the far-right scene in Poland. We were sitting in a Warsaw café, talking about the aims of Krystian’s movement. The main arguments that Krystian and Ula advanced were concerned with the errors in contemporary attempts to categorize present-day movements as right- or left-wing. Ula claimed that the classic division of political struggles along two axes — economic and cultural — was insufficient. It was not enough, she claimed, to say that a movement or a party could be economically liberal but morally conservative. This two-dimensional picture no longer captures the diversity of socio-political phenomena. Krystian did not share her emphasis on culture, precisely for the reason that he rejected the Gramscian interpretation of Marxism that, in his view, also approaches culture as an autonomous sphere. But he did agree that the two-axes model is insufficient and suggested that, precisely for this reason, he found a “horseshoe” model of the political landscape much more convincing. According to this model, the radical right and the radical left occupy the ends of the horseshoe — and, consequently, are close to each other. Listening to him, I drew a picture of a horseshoe, asking him to place Polish political parties along the arc. He did so and, pointing to the area between the two ends of the horseshoe — that is, between the radical right and the radical left — he observed:

“From the perspective of your research, this area is the most fascinating.”

“You mean: the question would be ‘How to close the circle’?” I asked.

“No, the question is rather whether it should be closed,” he replied.

Anthropological wisdom suggests that ethnographers ought to follow their research participants. It is a conviction that informs my various research projects. In this article, I follow Krystian’s advice and attempt to investigate the political terrain between radical right-wing and radical left-wing ideologies by analyzing the activities of two youth movements that I have been studying in recent years. In depicting their activism, I demonstrate why Gramscian thought may help us to better understand the often surprising affinities between ideas professed by the actors occupying different sides of the political spectrum. Or, to paraphrase the above comment by Ula, I show why we should read Gramsci to better understand the increasing appeal of certain “left-wing” ideas among activists who increasingly reject the label “right-wing.”^1^

I want to make it clear, however, that it is not my aim to argue about the “increasingly blurred boundaries” between left and right, echoing numerous observers who use this argument to explain the contemporary political vacuum. On the contrary, what the movements I study reveal is a clear re-articulation of specific ideological positions that inspire their own projects. I also want to note that, although I use the adjective “far-right” when describing my research participants, I do so with reservation, recognizing that the term does not precisely render the agenda(s) that combine radical nationalism with socialist thought. On the other hand, these kinds of movements have a long history when we consider historical instantiations of the variously far, radical, and extreme right, first of foremost fascism and national socialism.

Bringing in history is important when we consider Antonio Gramsci as a companion in our discussion. Gramsci’s role in my article is threefold. First, since he was a keen observer of fascist developments (not to mention the fact that he was a victim of Italy’s fascist regime), I relate his observations on fascism and inquire into their relevance for understanding present-day developments. I am particularly interested in Gramsci’s reflections not about fascism per se, but about the reasons behind its success and the role of the left in this process. Second, I explain why I find it helpful to explore the agendas of the movements in question through the Gramscian lens. Inspired by the special issue’s editors, I examine the extent to which his concept of “common sense” is helpful for analyzing contemporary far-right activism. I consider how a “far-right” common sense emerges through heterogeneous experiences and understandings and I relate it to Gramsci’s reflections on culture. Third, I relate my own ethnographic observation to analyses of the broader terrain of far-right politics to shed light on the phenomenon of “far-right Gramscianism.” As my discussion demonstrates, Gramsci has had a profound impact on far-right milieus. For all these reasons, he is a particularly apt guide for investigating the “fascinating area” between the radical left and the radical right.

In the following, I discuss these three aspects, moving from Gramsci’s reflections on fascism through my own ethnographic material on “fascism*s* of our time” (Holmes 2019) to an overview of far-right activism, and far-right Gramscianism, more broadly. Bringing all these aspects together, in the concluding section I reflect on the Gramscian framework for an understanding of the present-day left–right dynamics.

## Gramsci and (neo)fascism

Writing about the reactions to the rise of Benito Mussolini in communist Russia, the historian Stanley Payne observed that “[f]ascism was sometimes perceived not inaccurately as more of a heresy from, rather than a moral challenge to, revolutionary Marxism” (1995: 113). He further demonstrates that Mussolini saw communist Russia as the only political system where revolutionary and ideological counterparts to fascism could be found. Importantly, such views remained prevalent among fascists, even after their defeat during the Second World War, an attitude made evident in declarations suggesting that “the truest fascists” would welcome Stalin’s communism rather than Churchill’s and Roosevelt’s “plutocracy” (Parlato 2001: 7–8). The point regarding similarities and commonalities can be extended much further, of course, and may include domains as different as art, aesthetics, language, or approaches to violence: in short, aspects that supposedly prove the revolutionary nature of fascism and communism.^2^ At the same time, it could be argued that such similarities and declared alliances are irrelevant given that fascist Italy and the Soviet Union were, “after all,” political opponents —a fact first demonstrated during the Spanish Civil War (1936–1939).

This latter approach, amplified by the events of the Second World War, became dominant as a result of Cold War dynamics, when both left and right tried to purge their ranks of “fascism,” or, even better, to cast the term at their political opponents. While Marxist scholars described fascism as reactionary rather than revolutionary, particularly for safeguarding capitalist interests, their opponents on the right promoted the idea that totalitarianism was a common denominator for both fascism and communism. In one way or another, fascism was perceived as a sort of political aberration and treated as a monolithic phenomenon.

That this approach is simplistic is aptly illustrated by Gramsci’s approach to the revolutionary movements of the early twentieth century. A prisoner of the Italian fascist regime that wanted to silence him, Gramsci had also been censored by Italian communists who wanted to similarly silence his criticisms of the Soviet Union’s internal policies (for instance, persecution of political opponents). As is well known, Gramsci developed most of his theories during his stay in prison; ironically, fascism created the conditions of possibility that produced a great Marxist theorist. Gramsci’s notebooks and letters demonstrate that imprisonment led him to re-evaluate the force of fascism, which he had initially underestimated. However, given that he died in 1937, Gramsci did not witness the end of fascism. As such, his outlook does help us to understand fascism in the way that many of my research participants *want to* see it, even if they may completely differ from Gramsci in their evaluation. That is, they aim to see it as a peculiar sociopolitical “project under construction,” which has had its worse and better phases. It is not my aim to summarize Gramsci’s thought here, which is a task that has already been pursued by scholars who have traced the evolution of Gramsci’s thinking on fascism, from viewing it as a transient phenomenon to granting it a revolutionary status (e.g., Roberts 2011; Adamson 1980; Mangoni 1976). Rather, I focus on explanations of fascism’s success that are important for contextualizing debates about the rise of the far right today.

In identifying the sources of fascism’s success, Gramsci saw it as a response to a series of interrelated crises. He talked about an “unresolved national crisis” and claimed that “fascism is the name for a profound decomposition of Italian society which could not but accompany the profound decomposition of the state” (quoted in Adamson 1980: 78). At the same time, he placed the Italian case in a broader context that emphasized the crisis of liberal society and the liberal state (Mangoni 1976). Further, he talked about the detachment of social classes from political parties, the fact that the former did not feel represented by the latter, and that the latter were unsuccessful (or simply too passive) in reaching out to the former. Crucial here is the left’s inability to work towards a stronger connection between the elite and popular culture, and more broadly: to reach to, understand and consequently transform popular culture. To Gramsci, such a transformation was the condition of socio-political change. In arguing that fascism managed to fill the void (see Mosse 1966; Griffin 2002),^3^ he insisted that fascism’s success was a result of the weakness and failure of the left, rather than of the power of the fascist alternative. To Gramsci, left-wing politicians ignored what he saw as fundamental for carrying out a successful revolution in Italy, namely the profound impact of the First World War on Italian society, including both discontent and new aspirations brought about by the postwar context. Contrary to them, right-wing revolutionaries actually understood this (Roberts 2011: 241). Gramsci also shared the belief in the importance of a “spiritual revolution” and people’s “common will” with some right-wing ideologues (ibid.).

This is not to say that Gramsci diminished the importance of the “base” in favor of “superstructure.” On the contrary, questions of material conditions and structural constraints remain as central as socio-political ideas: his idea of a hegemonic “historical bloc” indicates a coherence of the economic (material forces) and the political (ideology). Unable to provide a new hegemony, fascism is an example of “Caesarism.” Gramsci understood this as a force (whether a person or a collectivity) capable of asserting domination and temporarily restoring equilibrium, yet unable to bring about profound social change: a passive revolution or a “revolution without revolution” (Gramsci 1979: 250).^4^ “Organic crisis” persisted under fascism, as this did not address the major contradiction (capital/labor) characterizing Italian society and had to use coercion and ritualized politics in the context of an absent genuine consent. Fascism’s incapacity to constitute an actual hegemonic alternative, Gramsci emphasized, resulted from the heterogeneity that characterized both proponents of fascism and their followers. At the same time, he acknowledged the role of fascism against liberalism, understood not only in terms of economics but as a cultural framework preventing social actors from discovering their potential to shape their own worlds (Roberts 2011).

While scholarly assessments regarding the accuracy of Gramsci’s take on fascism vary, he clearly provides an original framework that enables us to understand both the failures and successes of fascism and communism/socialism, which, more broadly, makes evident his influential role as a scholar — observer and analyst — of state transformations (Mangoni 1976). Some observers of current developments — especially those eagerly evoking the “return of the 1930s” — may be tempted to draw parallels with the contemporary crisis of the liberal order, the divergence between the political class and their electorates, or even new incarnations of Caesarism. Similarly, his reflections on the importance of culture not only shed light on the “culturally strategic projects”^5^ of far-right actors struggling to challenge the presumed left-wing cultural hegemony, but also force us to approach liberalism — which proponents of the far-right, “integralist” agendas challenge — as “an austere cultural phenomenon” (Holmes 2010: 10).

However, equally important for understanding this kind of radical politics, whether in the present or in the past, are not Gramsci’s specific comments *on* fascism/communism, but the ideas that arose *from* his analyses of fascism and communism. I mean here his reflections on common sense and the role of organic intellectuals, including politicians, in recovering “good sense” from chaotic common sense; the related question of language as a medium; and the (de)naturalization of social order, through the transformation of culture, as a condition of social change. In what follows, I illustrate different ways in which we can use these Gramscian ideas in the analysis of contemporary far-right milieus. More specifically, I aim to show that an emerging “new” far-right common sense is made of very heterogeneous influences (including “heretic” Marxist ideas). Furthermore, I demonstrate that that we can use Gramsci’s reflections on organic intellectuals and revolution to investigate two aspects of far-right activism: how activists interpret their own action through what they take to be a Gramscian lens and in what way the Gramscian framework helps us to interpret their activism.

## Gramsci and the far right today

Since 2016, I have been conducting a research project on transnational cooperation between youth far-right movements, with a special focus on Italy and Poland (Pasieka forth.). What connects the studied movements is that they are critical of liberal democracy, argue in favor of national autarchy, and link the defense of “traditional family” with the “national/patriotic cause.” All of them are youth movements and place a strong emphasis on the communitarian aspects of their activism, presenting themselves as diametrically different from political parties, even though, as I show below, they engage in political agitation for far-right parties. As for social backgrounds, members — predominantly male — come from all walks of life, with manual workers and unemployed young people engaging in militancy along graduate students and well-off entrepreneurs. In this article, the protagonists of my ethnographic material are two movements. Although they do not work together, they offer a good representation of a set of concerns and forms of activism that characterized all the studied milieus and, simultaneously, make evident the importance of Gramscian reflection for the understanding of the far-right scene.

The Italian association Loyalty Action (*Lealtà Azione,* hereinafter: LA) has been active in Italy for over a decade and has been very successful in developing all across Italy, even if its stronghold is still the north of the country. The number of core members in the respective chapters varies from several people to several dozen; among them are numerous sympathizers who join LA activities occasionally. Hierarchical and clearly structured, LA is divided into sections — branches — responsible for specific tasks: social assistance (provided to Italian citizens only), care for the environment and animals, actions raising awareness about persecuted Christians, martial arts and sport, and historical politics. LA considers “cultural work” to be crucial and frequently organizes discussions, conferences, and guest lectures by “cause”-friendly journalists and scholars. Although proudly identifying itself as a “community” and “movement,” in recent years it has lent support to several Lega (Lega per Salvini Premier) politicians.

The Polish movement, Polish Labor *(Praca Polska,* hereinafter: PP) has been active since 2017 and is much less numerous and widespread than LA, while the latter has dozens of members in each region, PL has dozens of members in total, and contrary to the wide range of activities LA offers, PL focuses predominantly on economic aspects and develops and promotes the agenda for a new Polish nation-state, based on social economy, social solidarity, and ethnonationalism. It does so through publications, online and offline conferences, as well as social assistentialism. The following two sections shed light on all these aspects.

### Milan 2016/2017

Reno, one of the founders of LA, was one of the first activists I got to know in Italy. In his sneakers, jeans, and sweatshirt, he looked like my university friend rather than a “typical” right-winger. Reno graduated in foreign relations and his collaborators joke that he performs the role of “minister of foreign affairs” in the movement. He also works for his father’s company, meaning that he travels a lot, and is responsible for the mountain section of the movement, which promotes a “reconnection with nature” and the “valorization of the national cultural-natural landscape.” Generally, the movement’s key aim, as Reno explained to me, is to lead to “national rebirth” before Italy “gets totally destroyed by immigration.”

Unsurprisingly, immigration was one of the key themes of our conversations. Reno would always emphasize that he and his movement are not against immigrants, but against immigration policies. “It’s not that we don’t want them, they simply can’t be happy in Italy” as “their roots are elsewhere” was the leitmotiv of our discussions. He stressed the inhuman conditions in which migrants often worked, which constituted an “offense to human dignity.” The biggest problem with labor migration, in his view, is the fact that it leads to uprootedness which, in turn, prevents “human flourishing.” As he explained to me, human dignity flourishes thanks to the use of one’s resources — through one’s labor — for which people receive fair pay; thanks to the possibility of living with family and community; and through rootedness in a culture and a territory, one’s natural environment. Labor immigration precludes all of this, and thus is to be limited and controlled.

While we can read such a statement as providing sophisticated cover for xenophobic rhetoric, characteristic of the increasingly common discourse on “autochthony” (e.g. Mepschen 2016), his obsession with “roots” was likewise manifested on numerous occasions. He avidly reads history books and travels around Europe to trace the remains of the Roman Empire. His focus on history, not to mention his emphasis on national rebirth, led us to discuss his fascist inspirations. His claims matched perfectly with what I had heard before, and what I would hear later from other radical nationalists in Italy, Poland, and Hungary: fascism is a rich source of inspiration, but not fully adaptable to the current circumstances.^6^ The contemporary global moment requires other responses, which is what makes movements like his different from left-wing ones that are obsessed with the past. Fascism was future-oriented, forward-looking — and the future is LA’s aim.

To explain the current needs of the moment, Reno invited me to take part in one of their campaigns: food distribution carried out in a peripheral district of Milan, a working-class area “forgotten by left-wing parties and elites.” One of the ethnographic visits I remember best was the distribution of special Christmas packages in 2017. Such a distribution takes place every month at one of LA’s headquarters, but what was exceptional that time was the fact that it took place outside. The activists chose a place in front of a church to put up their tent with tables, leaflets, and food packages. In fact, they had attempted to set up next to the church’s entrance, but the priest refused to give them permission. “Can you believe it? A priest!” one of the activists told me, emphasizing that he and his companions are discriminated against, even when doing “good things,” and even by a priest who should support charitable acts.

A dozen activists, mostly male, quickly distributed tasks for the day: some stood in front of the church, encouraging attendees of mass to stop by their stand, while others distributed packages to people they already knew who were scheduled to come. Others still collected information about families in need, promising to visit them at home and that, following an interview, they would begin delivering regular help. Due to the proximity to Christmas, people were offered a slice of *panettone* and a little prosecco. People who stopped by the stand lingered a bit to exchange a few words, lament their situation, and complain about corrupt politicians. Some left and came back again, in theory while taking their dog for a walk, but in practice apparently seeking further opportunities to talk. Others brought a friend or a neighbor who they thought would also benefit from regular assistance. The atmosphere was friendly; people exchanged gossip and Christmas wishes, and gathered together despite the biting cold.

We were standing there between a relatively new block of flats, the parish church, and a completely dilapidated public housing unit. At one point, Sandro, one of the LA leaders, invited me to join him and a journalist who had come to make a short video of the ghost-like building (as I later learned, the journalist had been following LA actions for a while). Apart from being a member of LA, Sandro is also a Lega representative and member of the local council. For him, walking through the rundown building is an opportunity to learn about urgent problems and to gain electoral support.

One of the inhabitants, a Romanian immigrant, served as our guide. She led us through the dirty, rubbish-filled corridors, illuminated only by a single bulb dangling from the ceiling. Some people agreed to talk to us, while others refused: those who did complained about “abusive” Roma people, about their own neighbors, about the inhabitants who had managed — “God only knows how” — to buy one of the municipal apartments. Some of them begin shouting at the Romanian lady and shut the door. The entire tour made it clear that the building was tormented by conflicts, grievances, and mutual accusations. Towards the end, we arrived at the apartment of an elderly lady whom Sandro already knew, and who was supposed to “tell her story” to the journalist. She invited us in and sat down with us while her husband remained in the kitchen; the apartment was perhaps 20 square meters in size. She recounted in detail the problems that she and the other inhabitants faced. The negligence of public authorities featured prominently in her account, including their disregard of Italians’ needs, low pensions, and drug dealers taking over a nearby park. She explained that her family had always voted left and that they were concerned with social justice. She clearly accentuated each word in a key statement: “I am a left-wing person” (*Sono-una-persona-di-sinistra*). Sandro seemed inattentive and scrolled through his phone, as if he already knew what she was going to say. His attention was piqued when the host praised the support provided by “good lads” (*bravi ragazzi*). When we were about to leave, the journalist asked her: “Don’t you mind that the people who are helping you are from a far-right organization?” The woman looked at her, amazed: “Are they?”.

On the way out, I tried to approach Sandro to ask him to help me understand the background to the problems that I had just learned about. He kept repeating that the situation in the building was “*incasinatissima*” – “the messiest possible.” I then asked him about the Romanian lady, as earlier on I had always heard that the LA only sought to support Italians, since, as they constantly emphasize, “every other organization and the Church only help foreigners.” His answer was not straightforward: he mentioned the woman’s helpfulness in understanding the situation, the fact that she had been in Italy for a long time, and the similarity of her “culture.” So I asked if the organization would help a Polish person like me if I came to Italy and was experiencing difficulty. He smiled and avoided a yes-or-no answer. One hour later, the food distribution was over and the activists got into their cars and rushed home for lunch.

A few months after the Christmas event, LA’s strategy proved successful, as a Lega politician whom they supported was elected to the regional government. In the period preceding the elections, which took place in March 2018, I witnessed increased campaigning during the distribution of food packages. Activists added electoral leaflets to the packages and Lega politicians were also to be present during food package distribution. However, only discussing LA activism in terms of political gains means neglecting a specific philosophy that informs their social assistance practices.

In proposing their clearly defined and carefully organized social assistance program, LA members challenge new models of welfare and volunteerism, which were thoroughly analyzed by Andrea Muehlebach (2012) in her discussion of the ways in which volunteerism and charity have become constitutive elements of “moral” neoliberalism. LA strongly criticizes the privatization of welfare, arguing that a well-functioning state should be in charge of activities undertaken by them in the state’s absence. They evoke a basic understanding of the social contract between citizens and the state, with the former having a *right* to work where they live that should be safeguarded by the state, which in turn ought to guarantee protection for those unable to sustain themselves. These ideas supplement the vision of human dignity and autochthonous natural rights that Reno outlined to me.

This is precisely where their wish both to represent “common sense” and to speak to the people’s “common sense” appear with great clarity. As Kate Crehan (2016) observes in her analysis of the American Tea Party,the aim of the movement is to frame economic issues and their proposed solutions into an emotive, convincing narrative that simply “stands for” common sense. LA’s strategy appears to be similar.^7^ The activists’ appeal to common sense, which “identifies the exact cause, simple and to hand” (Gramsci 1971: 419), translates in their case into a narrative on the state elites and left-wing politicians who are uninterested in Italian people’s lives, concerned only with migrants’ well-being, and, as such, to blame for the problems the inhabitants are facing. Instead, the discourse concerning the rights and priority of “autochthones,” including their own and their ancestors’ hard work, are presented as “natural” and “normal,” as reasonable and justifiable claims that “simply” represent social justice. As Crehan states elsewhere (2011), to Gramsci, “culture” is how the realities of class are lived. Such a culture, or such realities, are self-evident in the eyes of those who inhabit them. Aware of the targeted population’s sentiments, far-right militants appeal to what is considered as self-evident and natural *and* promise to help restore what appears to be “natural,” “just,” and “proper.”

Two kinds of activism appear particularly important in this undertaking. The first is the social assistance project described above that, as I noted, usually takes a different form. On the last Friday of the month, LA organizes an evening gathering at which the families they support receive their food packages. Those on the receiving end of such assistance socialize with one other, and may also be able to obtain information from a lawyer, learn how to write CVs from a social worker, or apply for social housing. During the Friday events that I attended, LA members mingled with guests and demonstrated a good knowledge of the clients they served, sometimes sharing common experiences while emphasizing the “common lot.”^8^ In the case of sick and elderly residents, the packages are delivered directly to their homes; this is an activity that LA members gladly document via Facebook and other social media to promote their actions. Whenever possible, however, they encourage assistance recipients to visit the headquarters, as meetings in such contexts are perceived as an opportunity to reinforce bonds between assistance givers and receivers and — in the electoral period — to campaign for befriended politicians. In short, the weight of the social assistance project does not lie in the actual quantity of help delivered — the packages LA prepares do not dramatically improve the situation of the families they support. Rather, the project gives far-right militants a chance to act as “organic intellectuals” whose aim is to articulate the disfranchised, neglected groups’ needs in a coherent form with the aim of proposing common-sense policies. It is also important to add here that while they are appealing to people’s common sense, their view of common sense is far from romantic. Like Gramsci, who at times expressed disdain for the “folk,” LA activists tend to express annoyance about the people’s ignorance and backwardness.

The social assistance project is part and parcel of LA’s broader agenda. The second key field of activism is diverse “cultural initiatives.” What I mean here is not activism concerned with “popular culture,” but rather an attempt to play a role in shaping the “elite” one. By organizing conferences and debates, publishing books and creating news platforms, LA engages in philosophical, historical, and anthropological arguments to offer an alternative way of thinking about issues as diverse as Plato’s criticism of democracy, heroism of Italian soldiers during the First World War, and the future of Europe. Although food package recipients are unlikely visitors to such events, cultural activism is meant to work in tandem with social assistentialism: both are supposed to repair what Reno describes as damage brought about by neoliberalism, “economic” and “mental” precarity. Further, its goal is to challenge left-wing hegemony in the field of culture and education: promoting activism in front of schools and universities, running a blog, and producing podcasts testify to the movement’s attempts to conquer new territories.

How unique is the LA agenda? Most far-right movements and parties of the fascist type have drawn extensively, even if inconsistently, on left-wing ideas. While the currently observed increasing popularity of “nationalist”-cum- “socialist” agendas may constitute an argument in a debate on the disappearing distinction between far left and right, the evidence that I present here suggests that something else is occurring. It seems that young people from LA, and numerous other kindred organizations, both in Italy and abroad, achieve more than simply attracting voters (for the far-right parties that they more or less openly support) or otherwise converting people to their political views. They are, no doubt, representatives of a new, “contentious European fascism,” whom Douglas Holmes recently (2019) described as proponents of a new “political imaginary which focuses directly on the substance of lifeworlds.” Thanks to “cultural work,” they successfully promote a “commonsensical” narrative on Europe’s “natural” ethnic composition and the need to defend the white autochthonous population, transforming exclusionary discourses into those featuring the societal order, justice, and deservingness. Yet does this turn their supporters into radical right-wingers and fascists? It seems, rather, that through a blending of discourses about culture and territory, about hard-working *folk* organically linked with their culture and territory, and about social justice and deservingness linked with belonging, they achieve something else: namely, their narratives and practices appear to be so successful as to make a Milan resident — a proud leftist — declare her support for those “good lads,” while maintaining her beliefs and political identity. Referring once again to Crehan’s analysis, we should thus recall that the struggle for hegemony does not mean “the creation of new narratives by new organic intellectuals, but rather the effective dissemination of already existing narratives, recrafted to resonate with the concerns of a given historical moment” (2016: 119). My Polish case study further supports this point.

### Warsaw/Katowice, 2020

When I first met Krystian, I quickly realized that I was unprepared: I had not read Thomas Piketty’s *Capital*, which he quoted in one of his first statements to me, asking if I remembered the story of the privatization of the Renault factory. He noticed that I felt embarrassed, and in order to cheer me up, said something like, “Oh, don’t worry, you specialize in ethnic minorities and religious pluralism,” which made me realize how well *he* was prepared for our conversation.^9^ As mentioned in the introduction, Krystian is the founder of the organization Polish Labor, which he established after leaving another far-right group*.* Within the last four years, Krystian has managed to gather several dozen activists and establish organizational structures in several Polish cities. Krystian and his girlfriend, Maria, are the leading figures in the new organization. Both are in their early twenties and combine their studies with work.^10^

PP shares with many other radical nationalist organizations the belief in sovereign nation-states, criticisms of the E.U. and the U.S., and the rejection of foreign capital. The nation, in their view, has an ethnic basis, and they believe that migration to Poland and Europe ought to be stopped. What makes them stand out from similar organizations is their very detailed social economy program, which includes advocating for progressive taxation, availability of social housing, widespread worker unions, and a reformed labor code. Although PP members do not remember the time of the Polish People’s Republic, they call for correcting the mistakes of the postsocialist transformation. For example, they consider it vital to address problems of regional marginalization by restoring bus and train connections, which were removed in the post-socialist era due to their presumed unprofitability, and to support the mining industry. Some of their claims appear contradictory if one considers, for instance, their emphasis on environmentally friendly policies and care for nature together with their emphasis on the necessity of building nuclear energy and bringing back heavy industry. Other policies, by contrast, may appear progressive in comparison with other far-right groups in Poland, as PP promotes religiously neutral policies and widely available nurseries and daycares that would allow both parents — mothers and fathers — to work.

PP promotes its ideas and puts them into practice through three forms of activism: “social campaigns” — which in fact mean “information campaigns”; “events” — usually one-day long conferences and fund-raising events; and “library” — publication and promotion of contemporary and historical works by authors who address national and social questions. Tech-savvy, PP activists successfully use social media to promote their activities. They stream most of their conferences and lectures online. They place a strong emphasis on design and aesthetics, making sure that the materials produced are high quality, eye-catching, and different from what they describe as “typical” nationalist propaganda materials — full of martyrological content and national-religious symbolism. Their logo includes a simple graphic of an ant, and they rarely use national symbols in their promotional materials.

Similarly to Reno, Krystian and his collaborators point to the problem of migration as one of the driving forces behind their activism. Krystian openly states his opposition to foreign laborers, whose numbers have increased in Poland in recent years (1.5 million Ukrainian citizens worked in Poland in 2019; a four-fold increase over 5 years).^11^ Yet, whereas other nationalist groups that I have been studying tend to talk about the actions of national governments or “E.U.” policies, Krystian emphasizes that “*capitalists* bring immigrants.”

He justifies his objection on several grounds. The first is a recognition that he shares with numerous other people in Europe and beyond: migrants mean competition and wages drops, and thus are not beneficial to the local (Polish) populations. The second is the fact that migration to Poland is not beneficial for Ukrainians: they are underpaid in Poland, and often work in scandalous conditions. He states that “Ukrainians are less and less satisfied with the work in Poland. Poles treat them as serfs.” The employers are eager to employ them because they are more subordinate and dependent on them. Similarly to LA activists, he sees the underpaid migrants in his home country as a local expression of a global phenomenon: the dominance of market logic. In order to raise Ukrainians’ awareness of this issue, PP created a poster targeting Ukrainian workers and informing them about the minimum wage in Poland to help them realize that they are underpaid and exploited. The third reason for his objection is that, by leaving Ukraine, migrants — usually young and skilled people — are slowing the process of change in and transformation of their own society. As a consequence, they are also undermining the process of global anti-capitalist change.

The Russian invasion of Ukraine in February 2022 did not alter Krystian’s approach. Contrary to other Polish nationalist movements which expressed solidarity with Ukraine, he continues to argue about the potential threat that the waves of Ukrainian newcomers pose to Polish society and the Polish economy. He presents a similar take on the ongoing protests in Belarus, strongly opposing the anti-Lukashenko protests, claiming that the worst possible fate for Belarus is to become “yet another Western colony.” Generally, ethnic/national diversity does not go hand-in-hand with working-class solidarity, Krystian claims. “Anti-capitalism needs to be global,” he says, but “Ukrainians [and Belarusians] need to do it [act against capitalism] at home.”

Krystian emphasizes that the movement is only taking its first steps and that they need to start a think-tank. “We need to arm activists with economic arguments,” he explains, “we need to *economize nationalism*.” Although the organ ization aims to express the interests of the working class, this is why it is mostly represented by students. He describes the organization’s member profile as “*inteligencko-pracowniczy*” (worker-intelligentsia) — a notion that calls to mind the discourse that was prominent in discussions on Polish anti-communist activism (see Kubik 1994). Krystian claims that the PP attracts two kinds of people: those with high cultural capital and a positivist attitude, and workers who are often union members. What unites them is the belief in “reformism” and in a gradual (step-by-step) revolution.

The people who attend events organized by PP tend to support this claim. One 2020 conference took place in Katowice, a mid-size city in southern Poland, in a small venue that they rented for the occasion. Among those in the audience were current and former students, as well as a few young men who seemed to have just left a football stadium. PP organizes such events quite frequently, each time choosing a specific theme. The Katowice event was devoted to the situation of workers in the Silesia region, today and in the past. The first part of the event focused on the history of the national socialist movement in the region. The second part took the form of workshop, the aim of which was to promote conversation about potential cooperation partners for PP. Activists and guests created a circle to facilitate the exchange of ideas and to emphasize the horizontal nature of the movement. The first group of potential partners discussed by the group were entrepreneurs. A specific goal in the case of this target group is to make them sensitive to the issue of workers’ rights as well as benefits for the national economy: paying taxes in Poland and employing ethnic Poles. The second group targeted as potential collaborators were local representatives of the governing right-wing populist party, Law and Justice (PiS). In emphasizing the desire to cooperate with local party members, the speakers assumed that they were less corrupt than high-level politicians and genuinely concerned about everyday matters. Some activists objected, however, emphasizing that cooperation could be difficult due to the clerical outlook of PiS politicians. Others challenged these claims, meanwhile, suggesting that PP members should not exaggerate the role of religious matters when it comes to cooperation in non-religious fields.

Taking the floor, Krystian emphasized that what matters more is PiS’s views on “social questions.” In concluding, he stated that he hopes their agenda will evoke more and more interest among left-leaning people, especially those who do not feel represented by those parties dubbed left-wing, but mainly preoccupied with “sexuality.” One of his companions added that anti-fascism is yet another obsession of such groups. Instead of tackling economic issues and real problems, they sit at university conferences and ponder whether or not something counts as fascism. He proposed, however, that they at least try to establish cooperation with students of economics. Yet another activist drew attention to a potential alliance with football fans, in what appeared to be an inclusive gesture towards some of the conference guests. In trying to engage them in discussion, Krystian asked these guests to recount something about the social and class background of football fans. They confirmed that most of them were working class. However, they did not seem eager to engage in discussion; instead, they were happy to take part in the martial arts training that concluded the conference.

Many of the activities carried out by PP might equally have been offered by a left-wing organization. They offer free school tutoring for children from needy families and legal advice in the areas of labor law, social assistance, and house rentals. What they do and claim is centered on the idea of work and on the plight of working people. What prompted many PP activists to act were precisely their different work experiences. When asked about his reasons for establishing a movement, Krystian explains: “Well, I got onto the job market — and realized something had to be done.” He might be a student and the self-proclaimed founder of a think-tank, but he works at the reception desk in a hardware store. “I meet *these people* every day,” he says, and by “these people” he is referring to underpaid construction workers (Ukrainian and Polish) and industrial laborers. He thus claims to know how to speak “people’s language” — the aspect strongly emphasized by Gramsci. As Crehan notes (2016: 36), “Organic intellectuals not only help to bring about fundamental social change through their ability to transform raw, inchoate experience into articulate, coherent narratives, as intellectuals, they themselves emerge out of that experience.”

Krystian also emphasizes what influenced his decision. When we talked in 2020, he observed that PiS had won the 2015 election thanks to a generous social program and promises of redistribution, but by now the party had abandoned strictly populist claims centered on social issues and security. However, their initially generous social spending stoked societal aspirations, and this is where movements like PP step in: “The demons like us come now,” he says with an ironic smile. (I’m curious how he would respond to Gramsci’s observation: “The old world is dying, and the new world struggles to be born: now is the time of monsters.”^12^) It would be stupid, he observes, not to embrace the opportunity and fail to push for their view of a national-socialist order. The 2020 pandemic opened yet further opportunities to promote the view of national solidarity among working people. Their activism mirrored that pursued at the time by LA.

In talking about such societal aspirations, Krystian simultaneously sheds light on the trajectories of PP members. It is not necessarily the case that the young people who support movements such as PP are unemployed or have no choice but to accept any job offer available. More common among them is underemployment — to work beyond one’s qualifications, whether in their home country or as seasonal migrant laborers in Western European countries. Many of them are fed up with being temporary workers for foreign firms, and disagree with the work conditions offered, for example, by multinational corporations. They are aware of the discrepancy between earnings in their home country and in Germany, France, or the UK. To substantiate his claims, Krystian quotes Piketty to demonstrate the persistent inequalities between Western and Eastern Europe, and highlights Poland’s quasi-colonial status. All these issues are the subject of his book, which further attests to his aspiration to perform the role of intellectual of the far-right scene.

## Left, right, and the theft of Gramsci

Given the popularity of “prompting” Gramsci to comment on various contemporary issues, from globalization to the “slow food” movement (and even inviting him for a meal — see the comic book *Dinner with Gramsci* by Constantini and Stamboulis), one may be tempted to ask him to comment on the contemporary far-right scene. Would Gramsci see in Krystian, Reno and other LA and PP militants “monsters” who come into being in a moment of instability, or would he rather see them as “organic intellectuals” who try to reach out to contemporary “subalterns”? Quixotic as it may be to try to provide a definite answer to this question, I dare to suggest that even if Gramsci rejected the LA and PP programs, even if he considered them too meshed up and contradictory (as he considered fascism to be), he would approach them in a less dogmatic manner than numerous present-day commentators, including his heirs, tend to do. “We are in the process of losing our foremost thinker of and on concrete historical scenarios, Antonio Gramsci, to a reactionary right-wing cause,” reads the opening line of the International Gramsci Society Newsletter from 1999. Right-wing Gramscianism, the note suggests, misunderstands and misappropriates Gramsci’s thought, especially his notion of hegemony: “a productive concept – ‘hegemony’—is being abandoned by progressive positions and revitalised by reactionary forces” (van Kranenburg 1999).

If I suggest that Gramsci’s position would differ, it is because his writing on fascism, and on the crisis of the interwar liberal order more broadly, makes evident his capacity to think beyond partisan lines. First, he frequently emphasized the weakness of the left-wing alternative — an observation that also aptly describes the present day. Worth recalling here is a point made bluntly by Krystian, who described the left as preoccupied exclusively with “identity politics” and the “search for fascism,” and unable, if not unwilling, to speak to average people. Krystian’s opinion is caricaturesque, but it does point to a problem which is more and more often brought up in diagnoses of the weakness of the left, and which is made by different observers, from conservatives to radical leftists. Second, Gramsci did acknowledge the political-philosophical of his opponents, such as Giovanni Gentile (key ideologue of fascism), finding inspiration in their thoughts on societal becoming and unity (see Roberts 2011). As I have shown, far-right militants are also keen to take ideas from the other side of the political spectrum — or from the other top of the horseshoe — a transfer of ideas which, to the best of my knowledge, rarely goes the other way (i.e., from right to left). To be clear, in highlighting this imbalance I neither evaluate such a “borrowing” (as a positive development, for instance) nor do I ignore the fact that the far right uses the “horseshoe theory” as a political strategy to legitimize their views.

Nonetheless, the question remains: how should we interpret the far-right’s borrowings of left-wing ideological conceptions and of what is considered as left-wing modes of activism? One might insinuate that the far right simply attempts to monopolize more territory, therefore strategically embraces the ideas of social justice, welfare or workers’ rights to appeal to various “left behind” (such as those we encountered in my ethnographic materials from Milan). Similarly, one might imply that it tries to attract young generations by presenting itself as a truly radical force opposing “the hated ‘bourgeois system’” and open to “unconventional counter-cultural lifestyles”.^13^ One might also suggest that this tendency indicates the far right’s ideological emptiness and lack of core ideology. Finally, one might venture that the far-right ideologues simply realized what Gramsci claimed so persuasively: that to win a hegemony means to have the cultural power, shape public debates, to influence education, run influential newspapers.^14^ In proposing such interpretations, however, it is important to bear in mind that there is no single far-right agenda today. Even Gramscian inspirations may mean quite different things: while some radical right-wing militants take Gramsci’s anti-capitalism seriously, others strip Gramscianism of its Marxist origins, focusing only on cultural hegemony.

All the above interpretations feature prominently in current debates on the far right. Inspired by Gramsci’s thoughts on fascism, I would like to propose one more: to approach the political agendas of movements such as LA or PP as “projects under construction,” a sort of political experimentation and a search for an answer to the problems here and now (cf. Adamson 1980). Quite tellingly, the key far-right “importer” of Gramsci’s ideas, Alain de Benoist, the founder of the intellectual–political movement Nouvelle Droite (“New Right”), has recently announced his support for *La France Insoumise*, a left-wing populist political party in France. More generally, we may read the Gramscian approach as appreciative of “attempts at change” and condemning indifference more than condemning political adversaries.

The latter points bring to mind G. M. Tamás’s essay *On Post-Fascism,* in which he emphasizes that “[t]he mere idea of radical change (utopia and critique) has been dropped from the rhetorical vocabulary, and the political horizon is now filled by what is there, by what is given, which is capitalism (…) What is the point of theoretical anti-capitalism, if political anti-capitalism cannot be taken seriously?” (Tamás 2000). What I find most convincing in Tamás’s essay is the emphasis on the idea of *change*, which does not need to translate specifically as an alternative to capitalism but which indicates a willingness to engage with different ways of thinking (even, or maybe especially, those one deeply disagrees with) and to consider change possible. Criticism of such a deliberate lack of engagement and of an aborted political imagination is something Gramsci, Reno and Krystian could likely discuss over dinner (heretical as this thought may be).

At the same time, in light of the evidence presented in this article, Tamás’s reflections provoke yet another question. In paraphrasing his query, we could ask “What is the point of *grassroots* anti-capitalism, if political anti-capitalism cannot be taken seriously?” Movements such as LA and PP claim to perceive the globalized economic order as the key enemy and neoliberal capitalism as a morally and culturally destructive system. Yet to be able to act and spread their agenda, they establish alliances with political parties, and end up cooperating and campaigning in the name of the political agendas they claim to challenge. Therefore, in concluding our conversation with Gramsci, it is plausible to assume that while appreciating their political experimentation, he might observe that — similarly to the fascist movements from whom they claim inspiration — they simply pursue a “revolution without a revolution.”

## Conclusions

Inspired by my ethnographic research, in this article I have argued that Gramsci’s ideas may help us to better understand the “fascinating arena” between left-wing and right-wing ideologies, a territory that the far-right movements I have described seem to be fighting for. What are the implications of this observation? I find it important to distinguish two kinds: those for social theory and those for understanding present-day political developments.

As to the first, I suggest that the “theft” of Gramsci by the far right, the debates about whom his ideas “belong to,” or what kind of phenomena we can explain using his scientific apparatus provoke an important question: what makes a good social theory? Is it its capacity to explain concrete socio-political phenomena, in a given time and place, or is it the possibility to stretch it and adapt it to new contexts, carving from it some categories that are “good to think with”? Gramsci’s thought has inspired numerous scholars and fields of studies, including postcolonial, subaltern and globalization studies. This multiplicity is no doubt owed to the heterogenous nature of Gramsci’s writings, which make them open to a variety of, often contradictory, interpretations. It is therefore no surprise that it has also inspired people on the right, and it is far from surprising that the most inspiring concept was that of common sense and its relationship with (potential) hegemony. Due to the emphasis Gramsci puts on its heterogeneous nature, on the fact it constitutes an aggregate of disparate, contradictory conceptions, the concept of common sense sheds light on both the reasons for the far-right success and its potential defeat.

However, I have also been suggesting that the “common sense” that left-wing and right-wing activists and ideologues refer to is not necessarily as different as we might think. This leads to the second point, that of the implications for our analyses of the present day. Whether they want to admit it or not, the radical left and radical right share important features, among them strong anti-Americanism, rejection of market economy and condemnation of the neoliberal model (in very broad terms), anti-Zionism, protectionist policies and the autarchy model. Recent responses to the Russian invasion of Ukraine illustrate the similarity of certain political standpoints well. If the left does not want to recognize it, it is because of “the fear of contamination,” as suggested by Giacomo Loperfido (2018) who draws on Mary Douglas’s seminal work to explain the persistence of “moral prejudice” in the left–right dynamics. Moving beyond this optic is necessary because the “contaminated” terrain between the two appears to be an area where some fundamental questions are currently being addressed: regarding rights of “autochthones” vis-à-vis “migrants,” the idea of “work” as a human right, retrenchment of welfare state, and understanding of what counts as “national culture” or what makes “national identity.” Recent years have shown that neither being indifferent to these questions nor denouncing them as “xenophobic” and “backward” brings about a solution.

Crucially, the realization of these affinities ought not to lead to some sort of “domestication” of the far right simply because they embrace some “progressive” ideas, because they have learned to talk about social justice and because some of them are genuinely concerned with the plight of their neighbors. Scholars conducting the ethnography of the far right have long warned about the danger of mistaking empathy for sympathy (Gingrich and Banks 2006). The same is true for the voices of those whom far-right actors claim to represent. As Annika Lems argues (this issue), “A Gramscian reading of common sense demonstrates that everyday meaning-making practices should not be romanticised. While they carry the potential for a more socially just, progressive world order, they can also be the seeds for a darker, exclusionary future.”

Rather, then, a recognition of these affinities should foster a more critical stance towards our own positions which we like to see as progressive, open-minded, and critical. To correspond with this portrait, such a position must entail an engagement with the ideas and practices we might consider despicable, to dare to ask uncomfortable questions and, to paraphrase Tamás (2000), to restore utopia and critique to the rhetorical vocabulary (see also Gökarıksel 2017). It is a position that matters scholarly and politically. When asked to comment on how to counter the present-day far right, scholars of fascism — its past and present forms — have noted that beating fascism means addressing those problems that fascism was trying to address in its perverse way. Gramsci made similar claims, recognizing fascism’s ability to respond to both specific societal aspirations and maladies. Not much theory is needed here. His is a very important, albeit a simple, observation; as a matter of fact, it is a rather commonsensical suggestion.

## Footnotes

1 As a matter of fact, it might be appropriate to use inverted commas to mark ideas or forms of activism as “left” or “right” throughout the article as my research participants distance themselves from such categorizations. I refrain from this choice, assuming that however imperfect these categorizations might be, they still allow us to map the socio-political arena in which my research participants operate.

2 I leave aside the relationship between socialism and fascism that has been addressed in many influential works on fascism. See, e.g.: Sternhell 1986; Parlato 2001.

3 George Mosse argued that fascism’s success can be explained in cultural, rather than strictly political or economic terms. His contributions and those of other fascism scholars are well summarized in Griffin’s (2002) seminal article on “the primacy of culture” in the study of fascism.

4 Gramsci discusses these two concepts throughout his notebooks. I am following here extracts from the notebooks in Chapter 8 in the Gramsci reader, edited by David Forgacs (1979).

5 See Rodseth 2018 for an inspiring discussion on Gramsci’s notion of culture and hegemony.

6 I discuss the movements’ relationship to fascism elsewhere (Pasieka 2022).

7 The case analyzed by Crehan differs in many important ways given the Tea Party’s neoliberal economic agenda. Nonetheless, the very strategy of the both movements’ activists is similar.

8 While I cannot address this issue at length, the class background and financial status of members varies dramatically. To use Gramscian vocabulary, we could say that some of the new “organic intellectuals” certainly do not originate from poor working-class backgrounds. But, as Crehan observes, “The organic character of intellectuals is not a question of the personal origins of individual intellectuals; it is determined, rather, by the role they play in the process of a given class’s production of knowledge” (Crehan 2016: 32).

9 Before our meeting, he had spent several hours reading my publicly available publications, especially those related to my doctoral project on the situation of ethnic and religious minorities in Poland.

10 Regrettably, I have not yet had the chance to talk to Maria.

11 Due to the ongoing war in Ukraine and Poland’s being the main recipient of Ukrainian refugees – some of whom “turn” into laborers – the newest data is hard to interpret. For some data on earlier years, see, e.g.: https://www.kyivpost.com/ukraine-politics/iom-poland-is-main-destination-for-migration-of-ukrainians.html (Last accessed: November 17, 2022).

12 Quoted in Žižek 2010.

13 Jeffrey Bale and Tamir Bar-On trace the New Right’s responses to the 1968 revolution.

14 One of the best examples is no doubt GRECE (Groupement de Recherche et d'Études pour la Civilisation Européenne), a think-tank established by Alain de Benoist in 1968.

## References

[CR1] Adamson Walter L (1980). Hegemony and revolution. A study of Antonio Gramsci’s Political and Cultural Theory.

[CR2] Crehan Kate (2011). Gramsci’s concept of common sense: A useful concept for anthropologists?. Journal of Modern Italian Studies.

[CR3] Crehan Kate (2016). Gramsci’s Common Sense: Inequality and its Narratives.

[CR4] Gingrich Andre, Banks Markus (2006). Neo-nationalism in Europe and beyond: Perspectives from social anthropology.

[CR5] Gökarıksel Saygun (2017). The ends of revolution: Capitalist de-democratization and nationalist populism in the east of Europe. Dialectical Anthropology.

[CR6] Gramsci Antonio (1971). Selections from the prison notebooks.

[CR7] Gramsci Antonio (1979). Selected writings, 1916–1935 (edited by David Fogacs).

[CR8] Griffin Roger (2002). The primacy of culture: The current growth (or manufacture) of consensus within fascist studies. Journal of Contemporary History.

[CR9] Holmes Douglas R (2010). Integral Europe.

[CR10] Holmes Douglas R (2019). Fascism at Eye Level. Focaal.

[CR11] Kubik Jan (1994). The power of symbols against the symbols of power: The rise of solidarity and the fall of state socialism in Poland.

[CR12] Loperfido, Giacomo. 2018. Neither left nor right: Crisis, wane of politics, and the struggles for sovereignty. In *Worldwide Mobilizations: Class Struggles and Urban Commoning*, eds. Don Kalb and Massimiliano Mollona, New York and Oxford: Berghahn.

[CR13] Mangoni Luisa (1976). Cesarismo, bonapartismo, fascismo. Studi Storici.

[CR14] Mepschen, Paul. 2016. The culturalization of everyday life. Autochthony in Amsterdam New West. In: Duyvendak et al., T*he culturalization of citizenship. Autochthony and belonging in a globalizing world*. Basingstoke: Palgrave Macmillan.

[CR15] Mosse George (1966). The genesis of fascism. Journal of Contemporary History.

[CR16] Muehlebach Andrea (2012). The moral neoliberal: Welfare and citizenship in Italy.

[CR17] Parlato Giuseppe (2001). La sinistra fascista: Storia di un progetto mancato.

[CR18] Payne Stanley G (1995). A history of fascism, 1914–1945.

[CR19] Roberts David D (2011). Reconsidering Gramsci's interpretation of fascism. Journal of Modern Italian Studies.

[CR20] Stamboulis, Elettra, and Gianluca Costantini. 2011. *Cena con Gramsci*. Padova: BeccoGiallo Editore.

[CR21] Sternhell Zeev (1986). Neither left nor right. Fascist ideology in France.

[CR22] Tamás, G. M. 2000. On post-fascism: How citizenship is becoming an exclusive privilege. *Boston Review* 25(3): 42–46.

[CR23] Van Kranenburg, Rob. 1999. Whose Gramsci? Right-wing Gramscism. International Gramsci Society Newsletter 9: 14–18. http://www.internationalgramscisociety.org/igsn/articles/a09_5.shtml. Accessed 15 November 2021.

[CR24] Žižek Slavoj (2010). A permanent economic emergency. New Left Review.

